# Considering the Other Half of the Gut Microbiome: Bacteriophages

**DOI:** 10.1128/mSystems.00102-19

**Published:** 2019-06-04

**Authors:** Corinne F. Maurice

**Affiliations:** a Department of Microbiology and Immunology, McGill University, Montreal, Quebec, Canada

**Keywords:** bacteriophages, gut microbiome, microbial interactions, microbial metabolism, replication cycles

## Abstract

Bacteriophages, viruses specific to bacteria, regulate bacterial communities in all known microbial systems. My research aims to determine how they interact with the trillions of bacteria found in the human gut. To do this, I apply a whole-systems perspective on both communities, considering bacteriophage replication cycles, bacterial and bacteriophage diversity, temporal dynamics, interactions, and responses to perturbations. Building upon ecological concepts and an array of independent and complementary techniques, I study the human gut from a microbial perspective. In contrast with bacteria, working with bacteriophage communities comes with many significant challenges, starting with the limited experimental toolkit and curated databases. Yet an increasing number of studies are pushing these scientific boundaries every day. In the human gut, future research that includes bacteriophages will lead to many exciting and warranted research avenues. From phage therapy and immunization to targeted drug delivery, the sky is the limit in my opinion.

## Abstract (precis)

Bacteriophages, viruses specific to bacteria, regulate bacterial communities in all known microbial systems. My research aims to determine how they interact with the trillions of bacteria found in the human gut.

## PERSPECTIVE

Despite the recent surge in research on the gut microbiota and its role for human health, most studies to date remained incomplete, as they did not consider the main agents of bacterial death and horizontal gene transfer in nature, namely, bacteriophages (“phages” for short). These viruses, which are specific to bacteria, control their abundance, diversity, and metabolism in all known microbial communities. Early estimates of phage abundances in the gut suggested they are as abundant as their bacterial targets and are very diversified (1, 2). Larger and more-recent studies using high-throughput metagenomics sequencing have since emphasized the high abundance and complexity of phage populations in the human gut, and yet their roles in this system remain poorly understood (3). Many outstanding questions remain about phages in the gut, mostly focusing on their role for human health. While recent evidence supports a strong therapeutic potential of phages against pathogens (4, 5), there are also reports of proinflammatory or deleterious effects of phages (2, 6, 7). Going forward, increasing our functional understanding of how the gut microbiome impacts human health will necessitate the inclusion of phages.

Data from this burgeoning active area of research show that bacterium-phage interactions in the gut are dynamic and can be altered by age, diet, disease, and medication (8, 9). Importantly, these alterations persist over time, are disease specific, and seem to contrast with those observed in other microbial systems. Here, I detail some of the phage-led issues that we are looking to address in the gut in the context of child development, health, and chronic diseases. The main hypothesis driving my research is that phages actively shape the diversity and function of gut bacterial communities. And how they achieve this is tightly linked to how they replicate, through lysogenic or lytic replication. We experimentally manipulate whole bacterial and phage communities from stool samples and monitor them with a combination of single-cell and “omics” tools, to draw conclusions about how these communities interact and their functions. Going forward, I envision microbiome-targeted therapies that would use the naturally occurring interactions between bacteria and phages to enhance host health and therapeutic success.

## LYSOGENY: AN UNREALIZED POTENTIAL

In the gut, phages are commonly found as prophages integrated in bacterial hosts, where they can contribute up to 20% of their host’s genome. Decades of research in other microbial systems support the hypothesis that lysogeny is a refuge for phages under conditions in which environmental or host metabolic conditions are challenging for lytic replication (3, 10). However, recent evidence in the human gut challenges this paradigm (11, 12). Bacterial lysogens have been described as “molecular time bombs,” with induction significantly altering the abundances and diversity of the bacterial and phage populations (3, 10). Induction is usually triggered by stress response pathways, reactive oxygen species, DNA damage, and altered metabolism of the bacterial host. This has many consequences for both communities: the production of new phages increases the phage predatory pressure and competition for bacterial targets, the lysis of bacterial lysogens frees new niches for competing bacteria, and the activated stress-response pathways can alter bacterial metabolism and the host immune response (3). In host-associated microbial communities, antibiotics are known inducers. Dietary fructose and short-chain fatty acids have also been shown recently to induce prophages from a well-characterized gut bacterial population (13). However, despite the reported prevalence of lysogeny in the gut, there is no current estimate of the portion of active or cryptic prophages, what gut-specific conditions might trigger their induction, or to what extent their induction contributes to the increase in the level of extracellular phages typically observed in disease (3, 14).

Building upon my previous work showing that common medication upregulates the expression of prophage induction genes (9), my team is currently exploring how common this is in healthy individuals. Working up from a collection of gut isolates to whole fecal samples and *in vivo* animal models, our goals are to determine how common prophage induction is in the gut, which are the bacterial and phage winners when induction occurs, and whether this changes with time, host disease, or other perturbations. In this way, we aim to provide a whole-systems perspective on alterations of bacterium-phage interactions and their consequences for the human host. Going forward, I am also interested in determining if the newly produced phage particles remain infectious and what their role could be for bacterial strain diversification in the context of health and disease.

## VIRUS-LIKE PARTICLES IN THE GUT: FREE TO INFECT?

It is widely accepted that, on average, phages outnumber bacteria 10 to 1 in microbial communities. In such cases, lytic replication prevails in shaping bacterial populations. In the human gut, this ratio seems to be lower and even reversed, suggesting distinct modes of interactions and ecological strategies between these two communities. An increasing number of studies demonstrated that these free phages are active players in human health: their diversity and abundance change in inflammatory bowel diseases (IBD) and type 1 diabetes and their presence in lung mucus could provide a first immune barrier to pathogens (referenced previously [3]). Some evidence suggests that the free phages in the gut are derived from induced prophages (1, 15, 16), and yet the phage diversity changes do not always match the bacterial diversity changes, suggesting that lytic replication or other underlying mechanisms are at play. For example, we still do not fully understand what drives the transition from the predator-prey dynamics characteristic of lytic replication in healthy children toward lysogeny and phage genome integration in healthy adults (3).

Irrespective of their numbers, these free phages exert predatory pressures on gut bacterial communities. Several teams are using lytic phages to limit the expansion of known bacterial pathogens, such as the pathogenic species Escherichia coli and Pseudomonas aeruginosa, among others (3, 17–19). The approach taken in my laboratory once again represents a whole-systems perspective, where we are determining instead the consequences of altered predatory pressure of free phages for gut bacterial communities. For these determinations, we experimentally separate gut bacteria from free phages and monitor changes in the two communities with a combination of single-cell and sequencing tools. We can then explore the consequences of decreased or increased phage-to-bacterium ratios or expose bacterial communities to phage communities originating from individuals with distinct health statuses. We are currently using this ecologically driven approach to determine the role of phages in child stunting and in adult ulcerative colitis. Preliminary data gathered so far indicate that these phages remain infectious and can alter gut bacterial communities in an age-specific manner. Importantly, this ecological framework provides functional information as to how phages can shape gut bacterial communities. Mining our data sets for specific bacterium-phage interactions will undoubtedly provide additional insight into the phage host range in the gut, as well as into bacterial resistance mechanisms. In addition, we hope to better understand whether the changes in phage populations observed in disease could drive those observed in the bacterial communities and therefore exacerbate some symptoms.

The human gut is home to one of the densest microbial communities that has evolved with the human host to provide it with numerous services, including key metabolic pathways. As such, applying and ultimately testing microbial ecology concepts will provide us with significant insight into the functional dynamics of this host-associated microbiota. There is power in considering humans as dynamic microbial ecosystems: concepts of intra- and interspecies competition, community resistance, resilience, biogeography, invasive species, and environmental variability and disruption all become relevant from a human health perspective, and the experimental frameworks at the interface between microbial ecology, engineering, mathematical modeling, and physics can be used to tackle these issues.

Currently, the biggest bottleneck in phage research is the sheer amount of unknowns about them, including their tremendous diversity, their host range, their stability, what regulates them, their safety (from a phage therapy perspective), their classification, etc., to which one must then add the complexity of the human gut microbiome. Yet an increasing number of studies are pushing these scientific boundaries, and it truly is an exciting time to part of this movement. Over the next 5 years, I expect that animal- and plant-associated phage communities will become increasingly thoroughly characterized and that the insight generated will showcase their relevance for the health of their eukaryotic hosts. I envision that phages will become a prevalent research avenue to manipulate bacterial communities. Indeed, they are already strong contenders for the treatment of antibiotic-resistant bacteria. Going a (few) step(s) further, one could use them preventively to manage our bacterial symbionts and our immune response or to complement existing treatments. Phages might also provide insight into the physiology and evolution of their eukaryotic hosts, as we uncover the mechanisms allowing the high levels of lysogeny in host-associated microbial communities. As we gather more information on these fascinating members of our body, we will see that we live not in a microbial word but rather in a phage world.
